# CRISPR-Mediated Knockout of Long 3′ UTR mRNA Isoforms in mESC-Derived Neurons

**DOI:** 10.3389/fgene.2021.789434

**Published:** 2021-12-17

**Authors:** Bongmin Bae, Pedro Miura

**Affiliations:** Department of Biology, University of Nevada, Reno, Reno, NV, United States

**Keywords:** alternative cleavage and polyadenylation, CRISPR, 3′ UTR, embryonic stem cells (ESC), neuronal differentiation, long-read sequencing

## Abstract

Alternative cleavage and polyadenylation (APA) is pervasive, occurring for more than 70% of human and mouse genes. Distal poly(A) site selection to generate longer 3′ UTR mRNA isoforms is prevalent in the nervous system, affecting thousands of genes. Here, we establish mouse embryonic stem cell (mESC)-derived neurons (mES-neurons) as a suitable system to study long 3′ UTR isoforms. RNA-seq analysis revealed that mES-neurons show widespread 3′ UTR lengthening that closely resembles APA patterns found in mouse cortex. mESCs are highly amenable to genetic manipulation. We present a method to eliminate long 3′ UTR isoform expression using CRISPR/Cas9 editing. This approach can lead to clones with the desired deletion within several weeks. We demonstrate this strategy on the *Mprip* gene as a proof-of-principle. To confirm loss of long 3′ UTR expression and the absence of cryptic poly(A) site usage stemming from the CRISPR deletion, we present a simple and cost-efficient targeted long-read RNA-sequencing strategy using the Oxford Nanopore Technologies platform. Using this method, we confirmed specific loss of the *Mprip* long 3′ UTR isoform. CRISPR gene editing of mESCs thus serves as a highly relevant platform for studying the molecular and cellular functions of long 3′ UTR mRNA isoforms.

## Introduction

Most mouse and human genes are subject to Alternative cleavage and PolyAdenylation (APA) resulting in the expression of mRNA isoforms with different 3′ ends (Hoque et al., 2012; Lianoglou et al., 2013). Most commonly, APA results in alternative length 3′ untranslated regions (UTRs) but does not change protein-coding sequence (Chen and Shyu, 1995; Sandberg et al., 2008). The usage of distal poly(A) sites is dramatically enhanced in the nervous system and results in mRNA isoforms with longer 3′ UTRs. This lengthening of the 3′ UTR in the nervous system has been observed across multiple organisms including *Drosophila*, zebrafish, mice, and humans (Smibert et al., 2012; Ulitsky et al., 2012; Miura et al., 2013).

Alternative long 3′ UTR mRNA isoforms have been found to be important *in vivo*. For instance, the long 3′ UTR isoform of *Bdnf* is necessary for transcript localization to dendrites, and in turn, for dendritic spine morphology and synaptic plasticity in mice (An et al., 2008). The long 3′ UTR isoform of *Calm1* is required for hippocampal neuron activation and proper C1 dorsal root ganglion (DRG) development in mouse (Bae et al., 2020). In *Drosophila*, loss of the *Dscam1* long 3′ UTR was found to severely impair axon outgrowth (Zhang Z. et al., 2019). These studies make it clear that the longer APA isoforms of at least some genes are necessary for nervous system development and function. However, it is less clear what molecular and cellular roles can be attributed specifically to longer, neural-enriched 3′ UTR isoforms. Long 3′ UTRs would generally be predicted to harbor regulatory cis-elements such as microRNA target sites, leading to regulation of translational control. However, new roles are emerging for 3′ UTRs (Bae and Miura, 2020), including regulation of subcellular localization (Tushev et al., 2018), regulation of upstream alternative splicing events on the same gene (Zhang Z. et al., 2019), and a scaffolding role in assembly of protein complexes as they are translated (Berkovits and Mayr, 2015).

CRISPR-mediated deletion of the distal poly(A) site has been established as an effective method to abolish long 3′ UTR isoform expression in both *Drosophila* and mice (Zhang Z. et al., 2019; Bae et al., 2020). However, to interrogate the roles of long 3′ UTR isoforms in relation to their impact on translational control, alternative splicing, and mRNA localization, a cultured cell system would have many advantages. What has been lacking to date is identification of a neuronal cell culture system that both is amenable to rapid gene-editing and also expresses long 3′ UTR isoforms at the levels observed in brain tissues.

Here, we show that mouse embryonic stem cell (mESC)-derived glutamatergic neurons (mES-neurons) exhibit robust expression of long 3′ UTR isoforms that closely resemble to the expression pattern in the mouse cortex on a transcriptome-wide level. We present a strategy for achieving CRISPR-mediated deletion of distal poly(A) sites in mESCs and demonstrate its effectiveness for deleting the long 3′ UTR isoform of the *Mprip* gene. CRISPR-mediated genetic manipulation in mESCs is fast and efficient, thus permitting the rapid exploration of the cellular and molecular functions of long 3′ UTR transcript isoforms.

## Materials and Methods

### Ethics Statements

Usage and disposal of biological agents were conducted according to the University of Nevada, Reno Institutional Biosafety Committee guidelines.

### Short-Read RNA-Seq Analysis

Public RNA-seq datasets from mESC (SRR645824 SRR645826 SRR645828) and mES-neurons (DIV7 for days *in vitro* 7; SRR645846 SRR645849 SRR645851) were used for APA analysis. Fastq files were mapped to the mouse genome using STAR 2.7. Mouse GRCm38.102 genome was used as reference. Binary bam files were visualized in IGV 2.4.17 (Robinson et al., 2011). For 3′ UTR lengthening analysis, QAPA 1.3.1 was used (Ha et al., 2018). Pre-built 3′ UTR annotation 1.3.0 was obtained from the QAPA github (github.com/morrislab/qapa). 3′ UTR fasta sequences were extracted using the annotation bed file and mouse mm10 genome fasta sequences. 3′ UTR isoform usage was quantified using Salmon 1.4.0 and the Salmon index generated using the 3′ UTR fasta file. Poly(A) site Usage (PAU), values were obtained from the Salmon quantification. Subsequent data processing was performed in R 3.6.0. From the QAPA output file, we selected the most distal Poly(A) site transcript by filtering for the rows with the maximum length of 3′ UTR for each of the genes to obtain the absolute distal PAU (dPAU) values. Non-APA genes with single poly(A) site were filtered out. An expression threshold of 1 TPM across all the samples was used. *t*-test was performed, and FDR adjusted *p*-values were obtained. The dPAU fold-change between mESCs and mES-neurons was calculated for each gene and a volcano plot was generated using ggplot geom_point. For alternative splicing analysis, sorted bam files were then fed into rMATS 4.0.2 to identify alternatively spliced cassette exons. Output file was filtered by FDR < 0.05 and |IncLevelDiff| > 0.2 to create a high-confidence spliced gene list. Gene ontology (GO) analysis was performed for biological process using topGO (Alexa and Rahnenfuhrer, 2021).

For correlation analysis, first, absolute dPAU values were obtained using QAPA. For this purpose, QAPA analysis was performed independently using mESC vs mES-neurons dataset or embryonic day 14 (E14) vs postnatal day 30 (P30) cortex dataset (SRR1805814 SRR1805815 SRR1805824 SRR1805825). Output files from each QAPA analysis contained dPAU values for mES-neurons or P30 cortex. Then, the dPAU values were arranged in a way that the mean dPAU for mES-neurons and the mean dPAU for cortex were merged into the same dataframe by gene name. Then a scatter plot was generated using ggplot geom_point, and correlation efficiency was calculated using stat_cor in R.

### Neuro2A Cell Culture

Neuro2A (N2A; ATCC Cat# CCL-131, RRID:CVCL_0470) cells were maintained in DMEM (Thermo Scientific 11965092) supplemented with 10% FBS (R&D systems S11150). For differentiation, 5 × 10^5^ cells were plated onto a 100 mm dish with DMEM supplemented with 2% FBS and 20 µM retinoic acid (Sigma Aldrich R2625). Media was changed daily until day 7 when neuronal-like morphology was confirmed by light microscopy and RNA was collected.

### Feeder-Free mESC Culture

Immediately after thawing, mESCs (ES-E14TG2a; ATCC Cat# CRL-1821, RRID:CVCL_9108) were kept on MEF (Thermo Scientific A34958, RRID:CVCL_RB06) feeder cells for two passages. Cells were maintained on 0.1% gelatin (Sigma EmbryoMax ES006B) coated cell culture dishes in mESC medium (high glucose DMEM with Glutamax (Thermo Scientific 10566016) supplemented with 15% FBS (Sigma F2442; tested for mESC culture), 55 µM ß-mercaptoethanol (Thermo Scientific 21985023), 1X MEM non-essential amino acids (Thermo Scientific 11140050), 1 mM sodium pyruvate (Thermo Scientific 11360070), and 1,000 U/ml LIF (Sigma ESG1107) at 37°C and 5% CO_2_.

### Differentiation to Glutamatergic Neurons

mESCs were differentiated to neural progenitor cells (NPC) and then to glutamatergic neurons as previously described (Bibel et al., 2007; Hubbard et al., 2013). Briefly, 3.5 × 10^6^ cells were plated onto 90 mm bacteriological dishes in 15 ml NPC medium (DMEM with l-glutamine (Thermo Scientific 11965092) supplemented with 10% FBS, 1X non-essential amino acids, and 550 µM ß-mercaptoethanol). On day 2, cell aggregates were collected and transferred into a 50 ml conical tube and left at room temperature for 3–5 min until cell aggregates settled. Media was carefully discarded and replaced with new NPC media. The cell aggregates were gently mixed by pipetting up and down and returned to the bacteriological dishes. On day 4, media change was performed as described, and 5 µM of retinoic acid was added. Media was changed again on day 6 and day 8 with supplemented retinoic acid. On day 10, NPC aggregates were collected and dissociated with 1 ml of TrypLE (Thermo Scientific 12604013) at 37°C for 5–7 min. To halt the reaction, 8 ml of Trypsin inhibitor (Thermo Scientific R007100) was added. The NPC aggregates were gently dissociated by pipetting up and down and filtered through 40 μm cell strainer. Cell suspension was diluted in N2 media (Neurobasal (Thermo Scientific 21103049) supplemented with 1X N2 (Thermo Scientific 17502048) and 2 mM glutamine (Thermo Scientific 25030081) at 3 × 10^5^ cells/ml. Ten mL of cells were plated onto PDL (Sigma P7280) coated 100 mm cell culture dishes. Complete media change was performed at 4 h (day 10) and 24 h (day 11) with N2 media. At this point, short neurite extensions were visible. On day 12 (equivalent of neurons DIV 2) and 14 (DIV 4), media was replaced with B27 media (Neurobasal supplemented with 1X B27 (Thermo Scientific 17504044) and 2 mM glutamine). Cells were maintained until day 17 (DIV 7).

### RNA Extraction and RT-qPCR

RNA was extracted from cultured cells using the Trizol (Thermo Scientific 15596018) method. RNA was quantified using Nanodrop spectrometer. Five µg of total RNA was treated with Turbo DNase (Thermo Scientific AM1907). Then, 1 µg of DNase-treated total RNA was reverse transcribed using Maxima reverse transcriptase (Thermo Scientific EP0742). The first strand cDNA reaction was diluted 1:5 before performing RT-qPCR. RT-qPCR was performed using SYBR Select Master Mix for CFX (Thermo Scientific 4472954). BioRad CFX96 real time PCR machine was used and results were analyzed using the ΔΔCt method. Primers used for RT-qPCR are found in Supplementary Table 2.

### sgRNA Design

Guide RNAs (sgRNAs) were designed using CRISPick GPP sgRNA designer from the Broad Institute (Doench et al., 2016; Sanson et al., 2018; https://portals.broadinstitute.org/gppx/crispick/public) by providing DNA sequence for the region spanning the distal poly(A) site. In the case of *Mprip*, each output sgRNA was inspected assuring that one sgRNA targets upstream of the dPAS and one downstream, giving preference to higher “pick order” and “combined ranked sequences”. sgRNA oligos used for *Mprip* long 3′ UTR isoform (*Mprip-L*) knockout are found in Supplementary Table 2.

For bulk sgRNA analysis, the same CRISPick GPP sgRNA designer tool was used in bulk mode. Genomic coordinate bed file for the 150 bp upstream of the most distal 3′ UTR end was generated using the QAPA dPAU file as a reference. Bed file for the 150 bp downstream region was generated in a similar way for each of the genes. Then DNA sequences were extracted in fasta format using bedtools getfasta and GRCm38 mouse genome. Upstream and downstream fasta sequences were separately inputted for sgRNA design 500 sequences at a time. Only the top rank sgRNA was used for further analysis. Upstream and downstream sgRNA cut position information was used to estimate the expected deletion size for each gene. Data processing and plotting was performed in R.

### Construction of Plasmids

sgRNAs oligonucleotides were synthesized with CACCG at the 5′ end followed by the sgRNA sequence. The complementary oligonucleotide contained 5′ AAAC and 3′ C sequence at each end. Oligonucleotides were phosphorylated (10 µM oligonucleotides 1, 10 µM oligonucleotides 2, 1X T4 ligation buffer, and 0.5 µl T4 PNK in 10 µl reaction) at 37°C for 30 min. Then the oligos were annealed by heating at 95°C for 15 min and gradually cooling down to room temperature in a thermocycler. The pX333 plasmid (RRID: Addgene_64073), which contains duplex U6 promoter and sgRNA scaffolds, was used for dual sgRNA cloning. The first sgRNA (for instance, the sgRNA targeting the upstream of the dPAS) was cloned into BbsI digested pX333 by standard ligation (50 ng digested pX333, 1 µl of 1:250 diluted oligo duplex, 1X Quick ligation buffer, and 1 µl Quick ligase (NEB) in 10 µl reaction). Ligation reaction was transformed in DH5α competent cells. Successful cloning was confirmed by Sanger sequencing. Subsequently, the second sgRNA was cloned into BsaI site following the same steps. Final sgRNA plasmids were confirmed by Sanger sequencing.

Donor plasmid harboring the antibiotic resistant cassette was constructed using pUC19 plasmid backbone. The entire backbone was PCR amplified using KOD Xtreme Hot Start DNA polymerase (Millipore 71975). 750 bp upstream to the left Cas9 cut site and 750 bp downstream to the right Cas9 cut site were PCR amplified from mESC genomic DNA and used as the homology arms. The upstream/forward primer for the left homology arm and the downstream/reverse primer for the right homology arm contained one of the sgRNAs and its PAM sequence overhang. The 20–25 nt overlap sequences necessary for HIFI assembly were added by a second round PCR using purified first round PCR product and primers with overlapping sequence overhangs. The neomycin resistance cassette was PCR amplified from pEN759 (kindly provided by Dr. Benoit Bruneau) using primers with overlap sequence overhangs. Each fragment was assembled into the pUC19 backbone using HIFI (NEB). Primer sequences are found in Supplementary Table 2.

### mESC Transfection and Clonal Analysis

Four hours prior to transfection, 2 × 10^5^ cells were plated onto a single well (35 mm well) of gelatin-coated six well plate in mESC media. After 4 h or once the cells are attached to the plate, 2.5 µg of donor plasmid and 0.5 µg of sgRNA plasmid was mixed with 9 µl of PEI (Polysciences 23966) in up to 200 µl of Opti-MEM (Thermo Scientific 31985062) followed by 10 min incubation at room temperature. Transfection solution was dispersed drop by drop onto the cells. The plate was returned to incubator until the next day. On day 1, full media change was performed. On day 2, appropriate antibiotics were supplemented with fresh media. On day 3, surviving cells were lifted with 1 ml TrypLE (Thermo Scientific 12604013), diluted 1:100–1:200 and plated onto gelatin-coated 100 mm cell culture dishes. In other words, 100 µl is taken from the 1 ml cell suspension then plated into 10 ml mESC media to make a 1:100 dilution dish. Fifty µl out of the 1 ml cell suspension was plated into a 100 mm dish to make a 1:200 dilution plate. The remaining cell suspension can be diluted into 10 ml mESC media and plated as a backup. On day 4, isolated cells should be visible under microscope. Media with antibiotics was replaced every day. Dishes were kept for additional 5–6 days until defined mESC colonies were observed. mESC colonies were manually picked using micropipettes under microscope and transferred into each wells of a gelatin coated 48 well plate. Each clone was maintained for 4–5 days for cell expansion. After cells reached >60% confluency, they were dissociated with TrypLE by incubating at 37°C for 5 min. Half of the cells were used for genotyping and the other half were transferred into each well of gelatin coated 12 well plate for further cell expansion. Clones with the expected genotype were expanded to six well plates, and then to 100 mm dishes.

Genomic DNA was extracted by incubating the cells in 300 µl lysis buffer (100 mM Tris pH 8, 200 mM NaCl, 5 mM EDTA, 0.4% SDS) with proteinase K at 65°C for 4 h - overnight. Genomic DNA was ethanol precipitated and resuspended in 200 µl of molecular grade water. Genotyping was performed by PCR. Once confirmed by PCR, a large fragment spanning the edit region was PCR amplified and Sanger sequenced. Genotyping primers are found in Supplementary Table 2.

### Nanopore Sequencing

For full-length cDNA synthesis, SMARTer PCR cDNA synthesis kit (Takara Bio 634926) was used according to user manual. Total RNA was DNAse treated either on-column using PureLink DNase (Thermo Scientific 12185010) and PureLink RNA Mini kit (Thermo Scientific 12183020) or with TURBO DNase (Thermo Scientific AM1907). First strand cDNA was synthesized using 700 ng of DNase treated RNA, 3′ SMART CDS Primer II A and SMARTer II A TSO. cDNA was diluted 1:5 in water. cDNA was amplified by optimal (18–21) cycles of PCR using Advantage 2 PCR kit (Takara Bio 639137). For enrichment of cDNA for genes of interest, cDNA was enriched by capturing with biotinylated xGen Lockdown probes (IDT; probe sequences found in Supplementary Table 2) and xGen hybridization and wash kit (IDT). Target capture probe design tool was used for the design of 120 bp probes. Probes were selected based on the location (toward the 3′ end) and GC contents (preferentially between 40–65°C). Captured cDNA was purified using AMPure XL beads (Beckman Coulter) then amplified using Takara LA Taq DNA polymerase Hot-Start version (Takara Bio RR042).

For nanopore library preparation, captured and amplified cDNA was end-prepped (NEBNext FFPE DNA Repair Mix and NEBNext Ultra™ II End Repair Kit) and ligated (T4 DNA Ligase – NEB) with sequencing adapters (Nanopore SQK-LSK110). MinION Mk1B device and FLO-FLG001 flow cells were used for sequencing of the libraries. Minimap2 was used to map the reads by supplementing GRCm38.102 transcriptome bed file. Aligned bam files were visualized in IGV.

## Results

### mES-Neurons Express Neural Long 3′ UTR mRNA Isoforms

**FIGURE 1:**
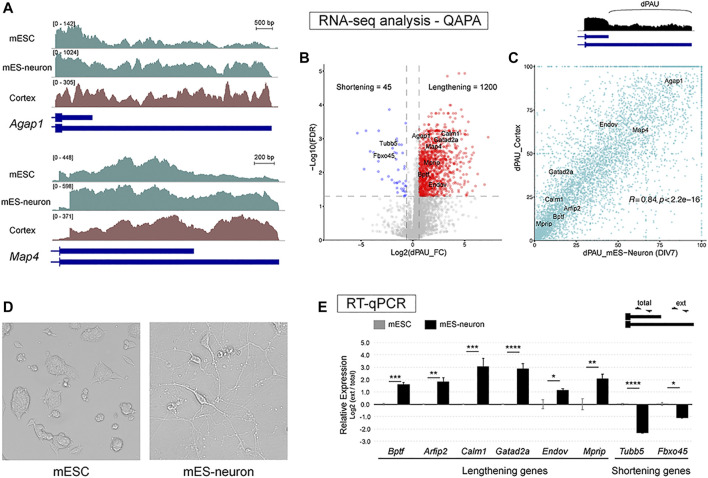
mES-neurons express neural long 3′ UTR mRNA isoforms. **(A)** Visualization of RNA-seq tracks for APA genes shows long 3′ UTR isoforms are upregulated in mES-neurons compared to mESC for two genes (green tracks). The expression in mES-neurons closely resembles mouse cortical transcript profile (brown track). **(B)** Assessment of distal Poly(A) site usage (dPAU) values derived from QAPA analysis of short-read RNA-seq in mESCs versus mES-neurons. Twelve-hundred genes showed significant dPAU fold-change increase from mESC to mES-neurons stage (Red). **(C)** Absolute dPAU values from postnatal day 30 cortex and mES-neurons were compared to assess their correlation. Comparison of dPAU values of all the APA genes between P30 cortex and mES-neurons showed a high correlation (R = 0.84, *p* < 2.2 × 10^−16^) suggesting that the long 3′ UTR isoforms expression strongly resembles the expression pattern found *in vivo*. **(D)** Image of mESCs and differentiated glutamatergic neurons days *in vitro* 7 (DIV7; mESC taken at ×10 objective and mES-neurons at ×20). Note the well-defined polarized morphology of mES-neurons. **(E)** RT-qPCR was performed for six lengthening genes and two shortening genes to confirm APA expression trends from the QAPA analysis. Long 3′ UTR isoforms expression was normalized to the total gene expression. Two-tail *t*-test was performed between the undifferentiated and differentiated cells; *n* = 3, * < 0.05, ** < 0.01, *** < 0.001, **** < 0.0001.

We first sought out a murine cell culture system that could recapitulate the long 3′ UTR isoforms that have been identified in mouse brain tissues (Miura et al., 2013). Mining public RNA-seq data we noticed that switching from short 3′ UTR usage to long 3′ UTR usage was evident when mESCs were differentiated into glutamatergic neurons for a handful of examples, including *Agap1* and *Map4* (Figure 1A). To assess whether this lengthening of 3′ UTRs during mESC neuronal differentiation occurred transcriptome-wide, we employed QAPA, a tool used to analyze APA. QAPA estimates the usage of different poly(A) sites for each gene from RNA-seq data and known 3′ end annotations (Ha et al., 2018). We were particularly interested in usage of the most distal poly(A) site (dPAU for distal Poly(A) site Usage), since long 3′ UTRs are more abundant in neurons. QAPA analysis showed a significant increase of dPAU in mES-neurons compared to mESCs for 1,200 genes, whereas only 45 genes showed the opposite trend (Figure 1B). GO analysis for the 1,200 genes with increased dPAU revealed these genes were enriched in biological functions including regulation of synaptic vesicle exocytosis, regulation of membrane potential, regulation of translational termination, and presynaptic modulation of chemical synapses. GO analysis for the 45 genes with decreased dPAU showed enrichment in GO terms such as, regulation of glutamine family amino acid metabolic process and smooth muscle cell proliferation (Supplementary Table 1). We next wanted to determine whether these APA events were correlated with changes that occur during mouse cortical development. We performed QAPA analysis on P30 cortex RNA-seq data to obtain absolute dPAU values. Comparison of dPAU values of all the APA genes between P30 cortex and mES-neurons showed a high correlation (R = 0.84, *p* < 2.2 × 10^−16^). Thus, mES-neurons closely resemble the distal poly(A) site expression patterns found *in vivo* (Figure 1C).

To confirm this lengthening trend with an independent experimental method, we performed mESC differentiation into glutamatergic neurons using established differentiation protocols with some adaptations (See *Materials and Methods*; Bibel et al., 2007; Hubbard et al., 2013). Feeder-free mESCs were cultured in suspension for formation of cell aggregates, during which retinoic acid was supplemented to induce formation of NPCs. Retinoic acid is used to halt proliferation-associated signaling pathways and to switch from proliferation to neural differentiation stage (Janesick et al., 2015). After 10 days of NPC induction, the NPC aggregates were gently dissociated and grown for seven additional days. Neuron differentiation media contained factors such as transferrin and insulin, which commit the cells for their final differentiation fate and assist with neuronal survival (Bottenstein and Sato, 1985). After 7 days of neuronal differentiation, well-defined neuron-like polarized morphology was observed (Figure 1D). RT-qPCR was used to determine APA changes for eight target genes – six lengthening genes and two shortening genes. We confirmed a significant increase or decrease in the expression of long 3′ UTR isoforms in the mES-neurons compared to mESCs (Figure 1E). We also investigated whether differentiation to a neuronal-like fate for N2a cells also led to 3′ UTR lengthening. However, we did not observe significant changes in APA by RT-qPCR for the same genes (Supplementary Figure 1). Thus, mES-neurons appear to be a good system for studying the regulation and function of neuron-enriched long 3′ UTR isoforms.

### CRISPR-Cas9 Strategy for Long 3′ UTR Isoform-Specific Deletion

**FIGURE 2:**
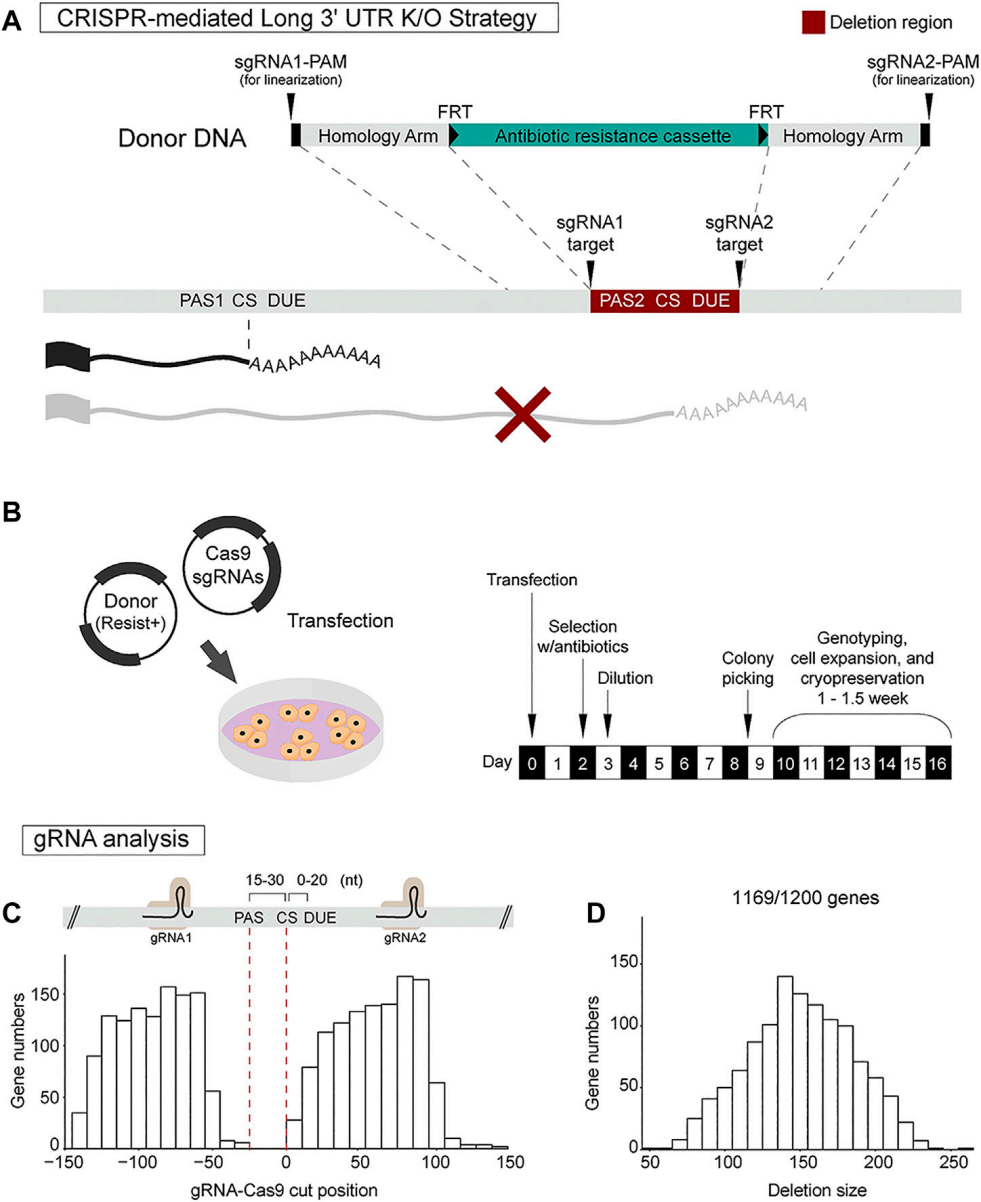
CRISPR-Cas9 strategy for long 3′ UTR isoform-specific knockout. **(A)** CRISPR-Cas9 strategy for long 3′ UTR deletion involves usage of a pair of sgRNAs to direct Cas9 and homology-directed repair (HDR) using donor plasmid harboring an antibiotics resistance cassette. The sgRNA pair targets regions flanking the distal poly(A) site. The donor construct for HDR includes sgRNA1 target site, protospacer adjacent motif (PAM) sequence, left homology arm, antibiotic resistance cassette, right homology arm, sgRNA2 target site, and PAM sequence. **(B)** The Cas9-sgRNAs plasmid and donor plasmid are transfected into mESCs. Antibiotic selection is initiated at day 2 post-transfection. On day 3, dilution of the cells is performed. Roughly 8–9 days post-transfection, mESC clusters are manually selected for further genotyping and cell expansion. **(C,D)** Analysis for designing dual sgRNAs at 300 nt genomic sequences flanking the annotated distal 3′ end of all the 1,200 genes (PAS, poly(A) signal; CS, cleavage site; DUE, downstream U-rich element). This analysis shows it is feasible to design dual sgRNAs for 1,169/1,200 genes (97.4%). On average, sgRNA pairs were located 90 ± 25 nt upstream and 62 ± 25 nt downstream of the 3′ end, introducing a deletion size of 151 ± 35 nt spanning the distal poly(A) site.

Previously, we established that CRISPR-mediated deletion of the distal poly(A) site is an effective way to abolish long 3′ UTR isoform expression in mice (Bae et al., 2020). We thus reasoned that a similar strategy could be applied in mESCs. sgRNAs are chosen that flank the distal poly(A) site to ensure that both the poly(A) signal and downstream U-rich elements (DUE) are deleted (Figure 2A). In most cases, a 100–200 bp deletion encompassing the distal poly(A) site can be designed. It is possible to delete larger regions of the long 3′ UTR sequence; however, distal poly(A) site deletion has been found to be sufficient in our experience and reduces the potential for disrupting potential DNA enhancers or silencers in the locus. In order to allow for efficient clonal selection, homology directed repair is used to insert an antibiotic resistance cassette at the deletion site.

Two plasmids are used for generating the deletion clones, a Cas9-sgRNA plasmid and a donor DNA plasmid. The donor construct uses pUC19 as a backbone and includes: 1) sgRNA1 target site and PAM (protospacer adjacent motif) sequence for donor DNA linearization; 2) 750 bp left homology arm; 3) antibiotic resistance cassette (e.g., neomycin) flanked by FRT flippase recombination sequences; 4) 750 bp right homology arm; and 5) sgRNA2 target site and PAM sequence for donor DNA linearization (Figure 2A). In this design, donor construct was flanked by sgRNA target sites and PAM sequences since the linearization of donor plasmid by sgRNA incorporation has been shown to improve homology directed repair in 293T and iPSCs (Zhang et al., 2017). Once co-transfected with Cas9-sgRNA plasmid, each end of the donor DNA construct is expected to be cleaved by Cas9 for linearization of donor DNA. The FRT sequences allow for future removal of the antibiotics resistant cassette which can be achieved by transiently expressing the flippase enzyme in CRISPR-edited cells (Gronostajski and Sadowski, 1985).

The Cas9-sgRNA plasmid and donor plasmid are easily transfected into mESCs using conventional transfection reagents such as polyethylenimine (PEI) which is a cost-effective cationic polymer that assists DNA transfection (Longo et al., 2013). Transfection of a single 35 mm well is sufficient to obtain multiple knockout clones. Antibiotic selection is initiated at day 2 post-transfection with a complete media change. On day 3, dilution of the cells is performed at a ratio of 1:100 or 1:200 into 100 mm dishes. Cells are maintained with constant antibiotic push. Roughly 8–9 days post-transfection, large mESC clusters are formed. The clusters are manually transferred into each well of a 48-well plate using a 200 µl micropipette. Within 6–7 days, sufficient cell growth is expected. Cells are gently dissociated, and half of the cells are transferred into each well of a new 12-well plate for expansion. The remaining half is used for PCR genotyping. Genotyping of 8–12 clones is usually enough to obtain homozygous knock-in lines (Figure 2B). Sanger sequencing is employed to confirm the precise deletion.

To determine how many of the 1,200 3′ UTR lengthening genes would be amenable to this distal poly(A) site deletion strategy, we extracted 150 bp DNA sequences upstream and downstream of each long 3′ UTR end and ran the sgRNA design tool to find the top rank sgRNAs pair for each gene. Our analysis showed it is feasible to design dual sgRNAs for 1,169 out of the 1,200 genes (97.4%). By plotting the sgRNA target sites relative to the distal 3′ end location, we found that that sgRNA pairs were located 90 ± 25 nt upstream and 62 ± 25 nt downstream of the 3′ end (Figure 2C). The expected deletion region size was 151 ± 35 nt spanning the distal poly(A) site (Figure 2D). This result suggests the robust applicability of the CRISPR strategy to generate long 3′ UTR deletions for nearly all genes of interest.

### Generation of Long 3′ UTR Isoform Deletion Lines for the *Mprip* Gene

**FIGURE 3:**
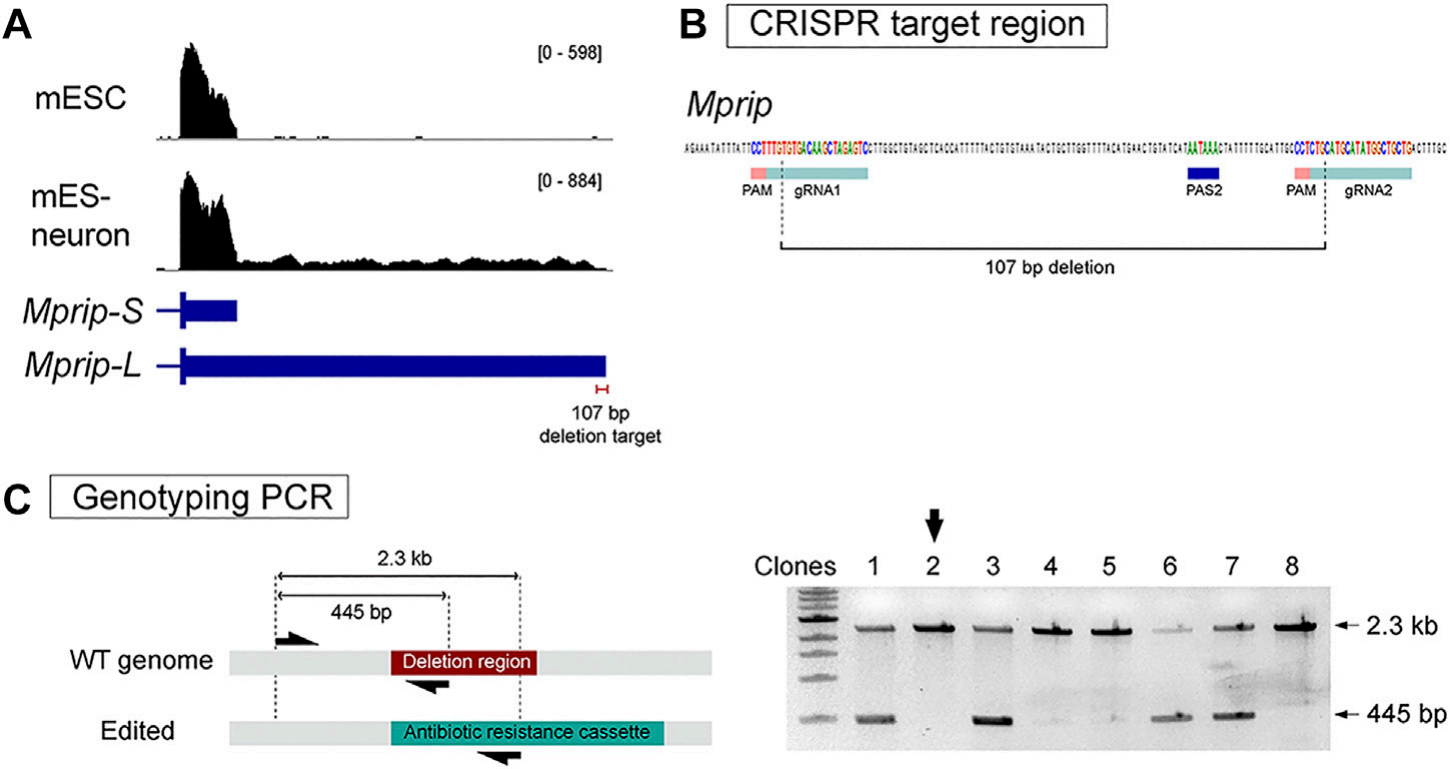
Generation of *Mprip* long 3′ UTR isoform knockout cells. **(A)** RNA-seq tracks show *Mprip* long 3′ UTR isoforms expression is increased during neuronal differentiation. *Mprip* generates a 650 bp short 3′ UTR isoform and a 5.2 kb long 3′ UTR isoform. **(B)** A pair of sgRNAs were chosen that flanked the distal poly(A) site. The strategy was designed to generates a precise 107 bp deletion. **(C)** PCR genotyping was performed using a three primer set – a common forward primer, a reverse primer specific for the target deletion region, and a reverse primer specific for the antibiotics cassette. Homozygous deletion clones were selected for further characterization (black arrow).

We applied our strategy to one of the 3′ UTR lengthening genes as a proof of principle. *Mprip* (Myosin Phosphatase Rho-Interacting Protein) encodes for a F-actin binding protein (Mulder et al., 2004). *Mprip* generates a 650 bp short 3′ UTR and a 5.2 kb long 3′ UTR (Figure 3A). QAPA analysis revealed that *Mprip* long 3′ UTR isoform (*Mprip-L*) expression is increased during neuronal differentiation (Figure 3A). RT-qPCR analysis revealed relative *Mprip* long 3′ UTR expression to be increased by ∼4-fold (Figure 1E). A pair of sgRNAs were chosen that flanked the distal poly(A) site. The 750 bp Homology arm regions were defined in accordance with the Cas9 cut site, which is 3 nt upstream of the PAM sequence. The strategy was designed to generate a precise 107 bp deletion (Figure 3B). mESCs were transfected and stable cell lines were generated as described above. PCR genotyping was used to select the clonal cells carrying the neomycin cassette (Figure 3C). Homozygous deletion clones were selected (Figure 3C; shown with black arrow) and propagated. The 107 bp deletion was confirmed by Sanger sequencing.

### Confirmation of Long 3′ UTR mRNA Isoform Loss Using Long-Read Sequencing

To confirm that the genomic deletion abolishes long 3′ UTR expression, differentiation into neurons is required. The preferred tool to confirm loss of a long 3′ UTR isoform is northern blot because both the expression level and length of the isoforms is revealed (An et al., 2008; Bae et al., 2020). In addition, northern analysis could also detect if spurious transcripts using cryptic poly(A) sites are activated by the genomic deletion. However, northern requires a large amount of starting material and thus is not convenient to apply on low expressed genes in mES-neurons. Thus, we present targeted long-read cDNA sequencing as an alternative approach.

**FIGURE 4:**
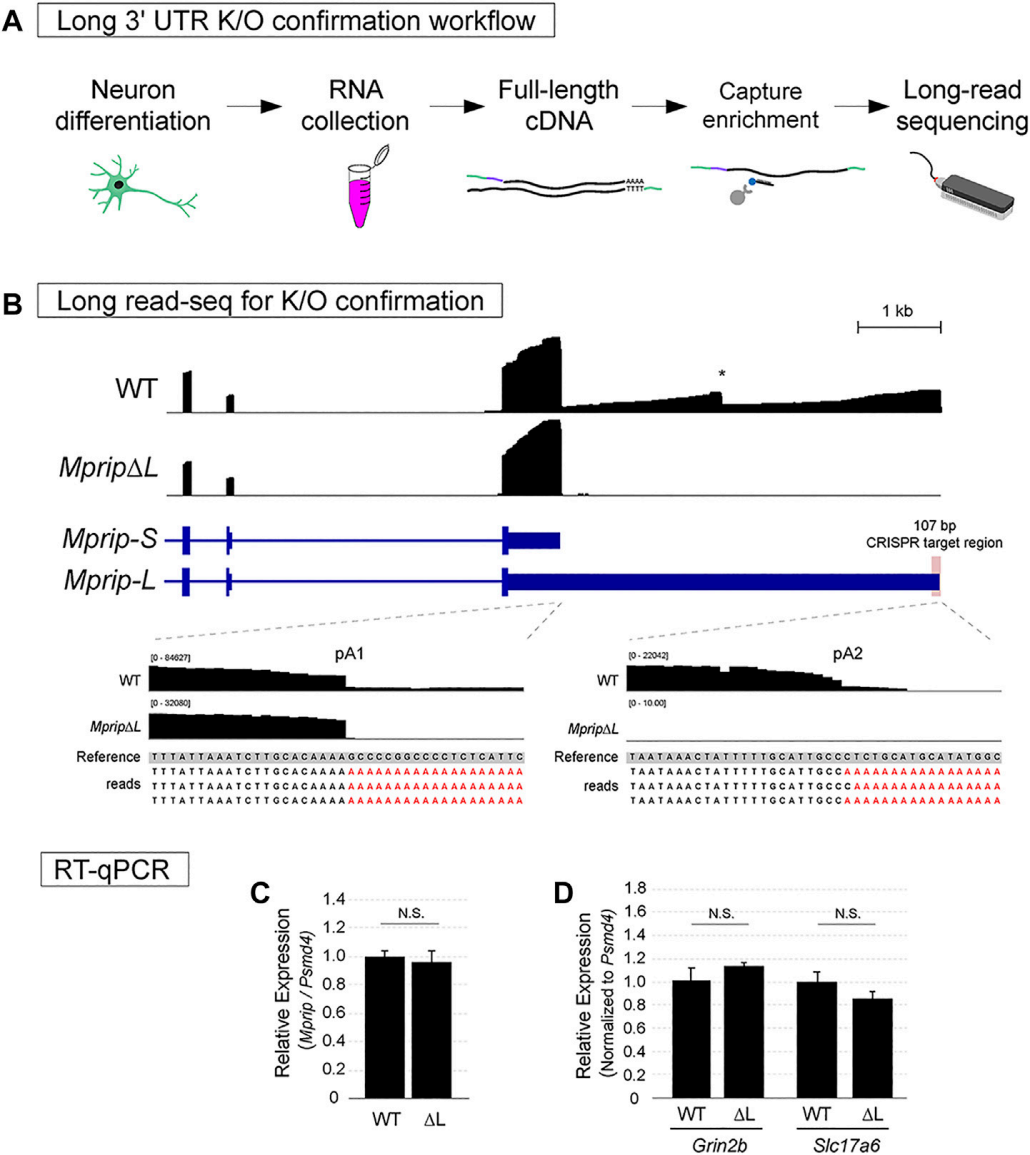
Confirmation of neuronal long 3′ UTR variant knockout using Long-read sequencing. **(A)** Workflow for assessing lack of the long 3′ UTR isoform expression. **(B)** Oxford Nanopore Technologies long-read sequencing data of WT and *MpripΔL* mES-neurons visualized in IGV. In the WT track, the two expected 3′ ends of *Mprip* and an internal mis-priming artifact were observed. In contrast to WT neurons, *MpripΔL* neurons lacked long 3′ UTR transcripts. No evidence of cryptic poly(A) site usage was found. **(C)** RT-qPCR was performed in order to obtain an accurate quantification of the total levels of *Mprip* in WT vs *MpripΔL*. The deletion did not alter the overall *Mprip* mRNA levels. Two-tail *t*-test; *n* = 3, N.S. = non-significant. **(D)** RT-qPCR for glutamatergic neuronal markers in WT vs *MpripΔL* neurons. *MpripΔL* neurons showed comparable levels of marker expression. Two-tail *t*-test; *n* = 3, N.S. = non-significant.

The targeted long-read cDNA sequencing approach relies on synthesis of template switching cDNAs and probe-based enrichment of the target prior to library preparation (Figure 4A). The template switching cDNA approach improves the synthesis of full-length cDNAs (Zhu et al., 2018). This cDNA synthesis relies on an oligo(dT)-based primer for detecting polyadenylated RNAs, thus allowing the recognition of 3′ ends. One of the limitations of conventional transcriptome-wide long-read cDNA sequencing is the difficulty in obtaining full length reads for low abundance genes (Byrne et al., 2017). Probe capture-based enrichment results in a library enriched for the gene of interest and is sequenced using the affordable Oxford Nanopore Technologies MinION platform.

To confirm loss of *Mprip-L* expression, WT control and CRISPR deletion cells were differentiated in glutamatergic neurons. Total RNA was extracted and full-length cDNA was synthesized. cDNA for *Mprip* gene and two other unrelated genes was enriched using xGen Lockdown probe pulldown. The library was prepared using Nanopore ligation sequencing kit and sequencing was performed using flongle flow cells on the MinION. Reads were mapped to the genome using Minimap2 (Li, 2018).

In the WT neuron long read sequencing libraries, the two expected 3′ ends of *Mprip* were clearly visible (Figure 4B; WT track). A drop off of read coverage was also evident at a genomically encoded A stretch in the middle of the long 3′ UTR (Figure 4B; WT track indicated by *). This is a typical artifact of sequencing methods that rely on oligo(dT) for reverse transcription. We confirmed that the reads did not contain untemplated poly(A) sequences at this internally primed false 3′ end. In contrast to WT neurons, the long 3′ UTR deletion neurons (*MpripΔL*) lacked long 3′ UTR transcripts (Figure 4B; *MpripΔL* track). There were no long reads aligning downstream of the deleted region which shows that cryptic poly(A) site usage was not activated by the distal poly(A) site deletion. This demonstrates that the deletion was effective in preventing *Mprip*-*L* mRNA expression. Genomic deletion of the distal poly(A) site might inadvertently dysregulate the total *Mprip* gene expression. In order to obtain an accurate quantification of the total levels of *Mprip* in WT vs ΔL, RT-qPCR was performed. This result showed that the CRISPR-mediated 107 bp deletion encompassing the distal poly(A) site did not altered the overall *Mprip* mRNA levels (Figure 4C). To rule out the possibility that the lack of *Mprip-L* expression in *Mprip*Δ*L* cells was due to impaired neuronal differentiation, we performed RT-qPCR for the glutamatergic neuronal markers, *Grin2b* and *Slc17a6* (Figure 4D). This result showed that *Mprip*Δ*L* neurons robustly express neuronal marker genes suggesting *Mprip*Δ*L* did not cause any neuronal differentiation defects. Altogether, targeted Nanopore long-read sequencing thus represents a rapid and accessible method for confirming long 3′ UTR mRNA isoform loss.

## Discussion

Despite the robust expression of long 3′ UTR isoforms in the nervous system, their functional relevance is underexplored. We have previously generated a long 3′ UTR isoform-specific knockout mouse for the *Calm1* gene (Bae et al., 2020), however, this approach is time-consuming and expensive. An *in vitro* system that recapitulates *in vivo* long 3′ UTR isoform expression might speed up the screening of long 3′ UTR isoforms for molecular and cellular functions. Here, we establish mESC-derived glutamatergic neurons as a suitable *in vitro* model for studying long 3′ UTRs. In comparison to the generation of a mouse line, abolishment of the long 3′ UTR isoform expression in mES-neurons was incredibly fast– allowing generation of extensive CRISPR-edited cell lines in a short period of time. Once 3′ UTR deletion mESC lines are established, mESC can be differentiated into multiple neuron types. Here, we presented an RA-mediated induction method of glutamatergic neurons, however, mESCs can be differentiated using alternative methods such as neurogenin expression (Reyes et al., 2008). Alternatively, mESC can also be differentiated into motor neurons (Wichterle and Peljto, 2008) or dopaminergic neurons (Friling et al., 2009). Exploration of long 3′ UTR isoform function in 3′ UTR deletion lines can thus be applied to different types of neurons.

In order to confirm long 3′ UTR isoform-specific deletion, northern blot has been a preferred method (An et al., 2008; Bae et al., 2020). However, northern blot requires a great amount of initial RNA and gene-by-gene optimization which might be inconvenient. RT-qPCR or short-read RNA-seq could be used to test for loss of long 3′ UTR. However, these techniques do not control for the possibility of spurious cryptic poly(A) site usage leading to spurious transcripts. Here, we devised targeted long-read sequencing as an alternative approach to circumvent the high RNA input requirement of northern blot analysis, and to ensure no cryptic poly(A) site usage resulted from distal poly(A) site deletion. This alternative approach demanded less than 1 μg of total RNA and provided information regarding the gene expression, usage of 3′ UTR isoforms, and cryptic poly(A) site usage.

This CRISPR-mediated long 3′ UTR deletion approach can open up new avenues to screen for long 3′ UTR functions. mRNA localization to subcellular regions of neurons can be influenced by 3′ UTR sequence content (Garner et al., 1988; Bassell et al., 1998; Moretti et al., 2015). Our understanding of 3′ UTR sequences that direct subcellular localization comes mostly from reporter based-experiments and genome-wide expression trends (Kislauskis et al., 1994; Tushev et al., 2018; Bauer et al., 2019). mES-neurons exhibit defined neuronal processes (Figure 1D), thus it should be feasible to detect subcellularly localized mRNAs using fluorescence *in situ* hybridization. Out of the 1,200 genes that showed robust lengthening of 3′ UTR in mES-neurons, 252 were found in at least one of two lists of hippocampal neuropil localized mRNAs (Cajigas et al., 2012; Tushev et al., 2018) (Supplementary Figure 2A). Whether sequences in long 3′ UTRs play a role in localizing these transcripts could be assessed using the methodology presented here.

Our method could be adopted to achieve proximal poly(A) site deletion of APA genes. This would force the exclusive usage of distal poly(A) sites to generate a “long 3′ UTR only” mutant cell line. Side by side generation of both proximal and distal poly(A) site deletion lines would allow direct comparison of the roles of each 3′ UTR sequence in subcellular localization. To accomplish this, the proximal poly(A) site region could be replaced by a donor sequence containing an FRT flanked antibiotic resistance cassette. Transient expression of flippase would complete the deletion of the proximal poly(A) site along with the antibiotics resistance cassette. In addition, our method, and another recently described approach (Mitschka et al., 2021), are amenable to deleting 3′ UTR sequences of single poly(A) site genes. To achieve this, our approach would have sgRNA1 targeted downstream of the stop codon and sgRNA2 targeted downstream of the poly(A) site. A donor DNA cassette would then be used to re-introduce a poly(A) site along with an antibiotic resistance cassette. Together, all these approaches facilitate widespread investigation into the functional role of 3′ UTRs in their endogenous genomic context.

Other reported 3′ UTR functions that have been shown for only a few genes, such as the RNA scaffolding role (Lee and Mayr, 2019) and the role in regulating alternative splicing (Zhang Z. et al., 2019), can be expanded to many genes using our technique. Alternative splicing analysis using rMATS (Shen et al., 2014) suggested that at least 135 genes that present lengthening of 3′ UTR during neuronal differentiation are also subject to alternative splicing (Supplementary Figure 2B). This provides a shortlist of genes to screen for long 3′ UTR dependent alternative splicing. Culturing of cells could easily be upscaled to obtain sufficient material necessary for molecular techniques such as mass spectrometry and ribosome profiling for studying the impact of long 3′ UTR loss on translational control. Although mES-neurons present limitations for modeling actual neurons in animals, they can be used to identify regulators of neuronal differentiation (Linares et al., 2015; Zhang M. et al., 2019), and to measure synaptic transmission properties (Whipple et al., 2020). In summary, our CRISPR-Cas9 strategy for generating long 3′ UTR isoform-specific knockouts provides a new avenue for the rapid exploration of long 3′ UTR functions at molecular and cellular levels.

## Data Availability

The original contributions presented in the study are publicly available. This data can be found here: ncbi.nlm.nih.gov/bioproject/776031.
