# Supplementary material for: CRISPR-Mediated Knockout of Long 3′ UTR mRNA Isoforms in mESC-Derived Neurons
Source: Front Genet. 2021 Dec 17;12:789434. doi: 10.3389/fgene.2021.789434 (PMC8718760; doi:10.3389/fgene.2021.789434)
Supplement: Supplementary file 1 [file DataSheet2.PDF]

Supplementary Table 1. GO analysis (Biological Process)

45 shortening genes

GO.ID	Term	Annotated	Significant	Expected	weightFisher
GO:0000820	regulation of glutamine family amino aci...	5	2	0.05	0.00098
GO:0048659	smooth muscle cell proliferation	42	2	0.42	0.00995

1200 lengthening genes

GO.ID	Term	Annotated	Significant	Expected	weightFisher
GO:2000300	regulation of synaptic vesicle exocytosi...	25	14	6.52	0.00013
GO:0007131	reciprocal meiotic recombination	13	9	3.39	0.00135
GO:0042391	regulation of membrane potential	89	34	23.2	0.00433
GO:0043628	ncRNA 3'-end processing	16	7	4.17	0.0046
GO:0051258	protein polymerization	101	29	26.33	0.0046
GO:0097050	type B pancreatic cell apoptotic process	6	5	1.56	0.00562
GO:0000963	mitochondrial RNA processing	6	5	1.56	0.00562
GO:0006370	7-methylguanosine mRNA capping	6	5	1.56	0.00562
GO:0006449	regulation of translational termination	6	5	1.56	0.00562
GO:0099171	presynaptic modulation of chemical synap...	6	5	1.56	0.00562
GO:0017145	stem cell division	16	9	4.17	0.00987
GO:0006342	chromatin silencing	27	11	7.04	0.00992
