# Supplementary material for: CRISPR-Mediated Knockout of Long 3′ UTR mRNA Isoforms in mESC-Derived Neurons
Source: Front Genet. 2021 Dec 17;12:789434. doi: 10.3389/fgene.2021.789434 (PMC8718760; doi:10.3389/fgene.2021.789434)
Supplement: Supplementary file 2 [file DataSheet3.PDF]

Supplementary Table 2. List of oligonucleotides used in this study

RT-qPCR primers	Bptf_uni_F;GCTGCCCTCATTTGTCAGCTA
	Bptf_uni_R;GGTCGCCTTGTACTTCACCTA
	Bptf_ext_F;CCATGTTAGCCCTGTCCTGC
	Bptf_ext_R;ATAGCAGGGCGACACCAAAT
	Arfip2_uni_F;AGTTCAACATCAAGCTGCGG
	Arfip2_uni_R;CGCCCAGTGTCCTTCTTGAT
	Arfip2_ext_F;ACCTGCACTGCTCTTGTTGA
	Arfip2_ext_R;TTGGACCTGTAGTTGACCGC
	Calm1_uni;CCTGCCCCTAAAGTCAAGC
	Calm1_prox_R;GCAAATGGAACTTTAAGGAGTCC
	Calm1_ext;TGTACTGTTGCCAGGGAAGG
	Calm1_ext_R;TGTTTAAGTTGCTCAAAGACACC
	Gatad2a_uni_F;CAGAGCTGGTTGCTGGGTTA
	Gatad2a_uni_R;TTGGCAGGCATGGGAATCAT
	Gatad2a_ext_F;GGTCTGACCAAGAGGGTTCG
	Gatad2a_ext_R;AACTGGGCCTACAAGGGTTG
	Endov_uni_F;GGCAACAGTGCATGGAACTC
	Endov_uni_R;GCATGGTGACCACACTGACTA
	Endov_ext_F;CAGGGGGAGGCAACCTAAAC
	Endov_ext_R;CCTGGCAGTCCACCATTCTT
	MpripL_F1;GCCTTCCCACTCATGCCATA
	MpripL_R1;GAGTCTGGGGTTTGGGTGTT
	MpripU_F1;AGGCACAGCAGCCTGTTAAT
	MpripU_R1;TCTGACCATCAAGGGTGGTG
	Tubb5_uni_F;CCCACGTCTCCACTTCTTCA
	Tubb5_uni_R;GGGTAAGTTCAGGCACAGTGA
	Tubb5_ext_F;TTTCTCGGAGAGATGGGGGT
	Tubb5_ext_R;CCCAAGTGTGGAGTTTCAGC
	Fbxo45_uni_F;TCCAGCTTAAAGGTGAGCGT
	Fbxo45_uni_R;TGTAAGATGGGACCGAGCCT
	Fbxo45_ext_F;CGCAAGAGAACACTGGGAGT
	Fbxo45_ext_R;AACGACACGTTTCCCTGGTC
	Slc17a6_F;CAATCATTGTGCCTATCGGGG
	Slc17a6_R;CATGCCAAAACCCCCACAAT
	Psmd4;CAGCCGATTCTTGGCTAAAC
	Psmd4;TCCAGACCTAAGCAGCATGA
gRNA oligos	BB811_Mprip;caccgGACTCTAGCTTGTCACACAA
	BB812_Mprip;aaacTTGTGTGACAAGCTAGAGTCc
	BB813_Mprip;caccgCAGCAGCCATATGCATGCAG
	BB814_Mprip;aaacCTGCATGCATATGGCTGCTGc
PCR primers	pUC19_F;GAATTCACTGGCCGTCGTTTTAC
	pUC19_R;AAGCTTGGCGTAATCATGG
	leftHA_1st_F;CCTTTGTGTGACAAGCTAGAGTCAAGAGATACCACTGTTAAGAATC
	leftHA_2nd_F;accatgattacgccaagcttCCTTTGTGTGACAAGCTAGAGT
	leftHA_R;AACCGGAAATCTCCACAAAGGAATAAATATTTCTACAAATTAACA
	Neo_F;ATATTTATTCCTTTGTGGAGATTTCCGGTTAAGAATTC
	Neo_R;GCAGCCATATGCATGAGTGGATCCATTATGTACCTG
	rightHA_F;CATAATGGATCCACTCATGCATATGGCTGCTGACTTT
	rightHA_1st_R;CAGCAGCCATATGCATGCAGAGGAGACAGGATTTTGCTGTGTAAC
	ightHA_2nd_R;AAACGACGGCCAGTGAATTCCAGCAGCCATATGCATGCAG
genotyping primers	BB4035_geno_F;GGCAGGGCCTCTTATATCTCG
	BB4036_geno_R;TGTAGAGAGAGCAGCAGGTTC
	BB4111_geno_R;CCAGCCTCTGAGCCCAGAAA
Lockdown capture probe panel	MPRIP_22_1;TCTTTTTGTTCCTTCAGACATAATGAAATCTAAAAGCAATCCTGACTTCCTGAAGAAAGACAGATCCTGTGTTACCCGGCAACTCAGAAACATCAGGTCCAAGGTGAGTGAGGTCCAGCT
	MPRIP_23_1;CTTATTTTTTGCTCTTTCCTCTGACAGTCCGTAATTGAGCAGGTCTCATGGGATAACTGAGTCGCGCCCGCTTCCAAGTCCCCTGTCGTATAGGTAAACCACTTCTCACTCTGCCCACTG
	MPRIP_24.1_1;TCTCCACCAGAGTCTGAAGGAAGGCCTGACGGTGCAGGAGCGCTTGAAGCTCTTTGAATCCAGGGACTTGAAGAAAGATTAGCTGCATCCATTCCACTGACACATCCTTCCCAGCTGGTG
	MPRIP_24.1_2;GCAGCACAAGCCAAGCGTGGTTTTCATCTTCACCTGTGCATCTCATTGTTGCTGTCATTAACTGTGGGGATGGATGCATGAGAGACTGCTGTGGGGTGCTTGCTCCAGTCCCTCCCTGTC
	MPRIP_24.1_3;AATTCCAACTTCCTGTTGTCTTTATCACAGCTTTTTTCCCATGGTATTCTCAGATTAGTCACGCGCAAGCAGTAGAGTGGGACGGTTTCCCCGCTGATACCTGAGCAGCCCCCTTGTCCT
	MPRIP_24.1_4;CCCAGCACACTCACTGTCAGTATTAAGGCACAGCAGCCTGTTAATAAGCTAGCCCTGCCTCAGAGTGCACATGTGGGGACCTGCCAGTGGCTCTGCTCTGCCGCACACAGCGGAGCACCA
	MPRIP_24.1_5;CCCTTGATGGTCAGAGCAGAGCCACCGAATGTTGTAGGCTGGCCTGATTTCTGAGGGGCCTAGGAAAGTGTCCAGCCTGGCCCACTTCCTTTTGATGTGGGAGGGGGACAGCATTTTGTA
	MPRIP_24.1_7;CCGGCCCCTCTCATTCCTTCCTCCGCTCGTGCCTCCCTTGCTGTGCTAATCCTGGTCTTCTGGTGGCCCGGAAGAGTGACTTGCAGCCTGAAGCTTCAGACTTGTCAAGTGTGATGGGTC
	MPRIP_24.1_8;CTCAAAGTCCTCTGGAGGGGAACATTGCTGGGGTTTAATCTGTCTGAAAAGAAGCTGCCATGCTGCTTGGCAGGGTGAGGAGAAGCCCGTGTGCAAAGACAGACTGTCAGGACTGGTGAT
	MPRIP_24.1_9;AGCAGCAGTGTGTGCTCAGGACCCTCGTTAGTCTGGGCTCTGGAAGGGTCCCTTGAATTGTCTGCTTCTCATGGAGCCATTGACAGGGAGCCTGTGCTAACTCTGCTTTTCTAGCTGAAC
	MPRIP_24.1_11;CCCATGTCAGATGGGAGAGGCAATAGCCTCATAGCTAGATATGCGATCTGGCTTCTTAGTCCTGGCTTGTTAGTGTTAAGTGGGTCAGATGGATGGTCTCTGAGCGTACAAATTCAACTC
	MPRIP_24.1_13;AGGCAAGCCCTGGGCTCCCCTTTTCACACGTTTTTAAGTTCCACTCTGGCACATGCTCAGTGGTCATTTATGTTTTCAGCATTATGGGCAAGTCAGACCTGTTAAAACAAGCTTTGAGCT
	MPRIP_24.1_15;CTGCCTTGTGCTTCCTGGGATGCTACAAAGAGCCTGCGGAAGATGCCAAGCCTACCATTCAAAAGGACACTTGATCTTTTGCTCTTCACCTCAGAATCCTAGGATATGAACTTTGCTGAA
	MPRIP_24.1_17;CTAGGGCTTGTTTCACGTGTGGGTATCTGGTATGTTGGCCAAATGCTGAACTGGCAGACATTCCTGGATCATCCCCCACTGTCTACGCCAGTCTGAGCAGTAACATGTGCAGGATCAGGG
	MPRIP_24.1_19;TCTGTCCATTCCCTTCCCTCACTCCCTGAGCCAGGTTCACTTTTGCCTGACCCCTCTACAGGCTTCCCATTCCCTGGAAAGAAGTTGAAGTTGGTGTGTTCAGAGAAGAGGTATGGAATG
	MPRIP_24.1_21;ACATTTGTGGTCTACCAGCATCTACAAGCAAACTACCATTACAGGGAACATACCATTTGACAGTGAATGTAAATATATGCTTAAATTACATCTTGCAAGCCTGGCATGGTGGTGCACGC
	MPRIP_24.2_1;AAAAAAACAAAAAAAAAAAACTTCATCTTTGCACGTGTGATGCCGAGCACATCTGCCACACAGGGCTACTGGCTGCACCTTTTCCAGTATGGTTGTGGCCACAATGCCACAATAATAGGA
	MPRIP_24.2_2;GGCTGCACCTTTTCCAGTATGGTTGTGGCCACAATGCCACAATAATAGGAGGCCAGAGCCCCATGAAGTAGGGAGTCCCCAGAGGGAAGAACGCTAGGAATGTGTGCATTCACTGAGTGT
	MPRIP_24.3_2;TTTGTCCAAAGGGAGCCTGAGAGATTTGACTAGGAAACAAATGGGTATGAAAGATATTTGAAGGAATGTGTACTACTGTTAACCTCTCTACAGGGGGAGATGGTGCGCTGACTTCTTTCT
	MPRIP_24.3_4;ATCAGCTTAGTTAGATTCCTGCAGGCCTGCCCCACACACCTGACCTCCAACTAAGCCTGGCTCTCAAAGACAGGCTGGATAAGCACCCAGAGGCCCTATGCAGCCTTCCCACTCATGCCA
	MPRIP_24.3_6;GTTACCTCTTGGCCAGCGGAGGGTTAGCTGTTCATGCTATGCTGTAAAGATGACATCACCTAGTAAGCTACTTAGAGGCTATCAGTGTAGGGACTCATACAGCCTTTCTCATTAGAGCTC
	MPRIP_24.3_8;TTTGTGCAAAGTTTGTGTGAGTTTTAAAATACCCCTTTTGAAGGTATGTAAAGGCAGTGTTCAGTGCAAACCCCTCTCAAATGTCAAGACCATTAGTTGGCGAGGCATGGTAGCACACAC
	MPRIP_24.4_3;CATACGCTGCTTCAGGTCCAGGAGCCTAGCTCTAACAGCGGGGCTGGACTTGAGTGCAGAGCAGCCCAGCTACCCATATCTCTAGCGACCTCACTGTATATAGCATGAGACTCCAGGTAG
	MPRIP_24.4_5;TGGAGCAGCTTCCATTGTGGGGCAGGACCTCATTGCTGGCATTTGCCATAAAGAGATACCACTGTTAAGAATCCTAGCCACACAAGCCCACTCCAGCTTGCCACAGCTGACATGCCATTG
	MPRIP_24.4_8;TAAGAACCGCAGTCACAGTGGCTCCTTGACAATCAGAACTCTAGGTGACCTTCCATTACTGCTGCTTGACTGGGGGTGGGGTGCGTGTTTGTGAACAGGGCGCCTGGCAGGGCCTCTTAT
	MPRIP_24.4_10;CCAGAGAGGCTTCAAGCACAGCTTCTCAGAGCCAGTTCAGGCCTCTGTGTGTATGCACAGGGTGTGGCTTCCTGCAGACAACATGTCCCCCAACTCCTCATTCCAAGATCTTATGGTTCA---
	AHCTF1_32_1;TCTGTCTGGTTTTCCAGCTGTGGCCAACACGGTTGCAGATGTTGAAAGCTCTGGGTCCACCAGCTCCAAGTGCCCGGTTACCTCTGAACGCAGCCTCGGCCAAAAACTAACATTAAACTT
	AHCTF1_33.1_2;TAATCTCAGTACTATTGAGCAGCAGTTTTGTGACTTGCCTGATGACAAAGACTCTGCTGAATGTGATGCTGCTGAAGTAGACGGGGAACTTTTTGTGGCCCAGAGCAACTTTACCCTGAT
	AHCTF1_33.1_3;TTTAGAAGGTGAAGAAGGAGAAGCTGAGGCAAGCGACTCTGCAGCACCTAATATGTTACCGAAAGCGACCAAGGAAAAACCTGTGTGCCACAGGGAACCCCATAATCAGGAGCGCGTTAC
	AHCTF1_33.1_4;AGATTTGCCATCTGCTGTGACTGCTGACCAAGAATCCCACAAGGTAGAGACTTTACCGTATGTGCCTGAACCGGTTAAAGTGGCAATTGCAGAAAATCTGTTGGATGTAATTAAAGACAC
	AHCTF1_33.1_5;CAGAAGTAAGGAAGCAACTCCCGTGGCAGCAGGTGAGGCTGGTGATGAGGACGGAGCAGTGATAGTCTCAAAGGCTGCACATTCGTCCAGGCTGACAAACTCTACACCGAAGACTGTTAA
	AHCTF1_33.1_6;GGAACCACATGCAGAGACTGTAAATACCAGCCAGAATGATGACATGGTTTCTTCTAGAACTCTCACAAGAAGGCAGCATGCCCTAAGCCTGAATGTCACATCAGAACAAGAGCCTTCAGC
	AHCTF1_33.1_7;AGTTGCCACTCCTAAGAAGAGAACTAGAAAAATTAAAGAAACTCCTGAGTCTTCTGAAAGGACCTGTTCTGACCTAAAAGTAGCACCTGAGAACCAACTGACAGCTCAGAGTCCTCCCGC
	AHCTF1_33.1_8;TCCTAGGAGAGGAAAGAAGAAGGACGTTAGCCAAGGCACACTGCCAAGTTCTGGTGCTGTGGAGCCGGAGCCGGAACCTCAGGGTACGCCGGGAAGACTGAGGCTGAGAACGCAGCCACC
	AHCTF1_33.1_9;CGAGCCAGCAGCTGAAGAAACTCCTTCTAGAACAAAAGTCAGGCTTTCATCTGTTAGAAAGGGAACCCCTAGAAGACTTAAGAAGTCTGTAGAAAATGGGCAAAGTACAGAAATTCTAGA
	AHCTF1_33.1_10;TGATCTCAAAGGGAGTGAGGCAGCAAGTCATGACGGGACTGTCACAGAGCTGAGGAATGCCAATTTAGAAGATACTCAGAATATGGAGTATAAACAAGATGAACACAGTGACCAGCAACT
	AHCTF1_33.1_11;GCCTCTAAAACGAAAGAGGGTCAGAGAGAGAGAAGTTAGTGTGTCAAGTGTGACAGAAGAGCCAAAGCTTGACTCATCCCAGTTGCCTCTTCAGACAGGACTCGATGTACCTGCCACCCC
	AHCTF1_33.1_12;TAGGAAACGTGGTAGACCCAGGAAGGTAGTTCCCTTAGAAGCTGATGGTGGCACAACTGGTAAGGAACAGACAAGTCCTCAGAAGAAAGATGTTCCGGTTGTCCGGAGATCTACACGGAA
	AHCTF1_33.1_13;CACCCCAGCTAGAAATGTGAGTACTTTAGAAAAATCAGTTTTAGTGCCAAATAAGGAAGCTGCTCTAGTGGTGACATCTAAGAGGAGACCTACAAAGAAGTCTGCAGAGGAAAGCTCAAA
	AHCTF1_33.1_14;AGATCCATCAGCGGCAGTCTCAGACCTGGCGGGTGGAGCAGCCCACACAGAGTCCGCTGACCGAAGGGACGGACTGCTTGCCGCCGCTGCTCTCACGCCATCTGCCCAGGGCACAAGGAC
	AHCTF1_34_1;AAACCAGACATAGTTACAGGTCGATGTTTTGTTTGTTTCCTGCTTGTCTTTAGCTGAGAACATGGAGGCCGCAGCCCAGCTGAAAGAATTGGTGTCAGATTTATCTTCTCAGTTTGTTGT
	AHCTF1_34_2;TTCCCCTCCTGCCTTGAGAACCAGGCAGAAAAGTATATCCAATACTTCCAAGCTTCTAGGTGAACTGGTAAGGAAGGCATGAAAACATCTCTGTGATTATTGAGTTATTTGTTTGTGTGT
	AHCTF1_35.1_1;TTTTAAATTTCTAACCCATAGGAGAGTGACCCTAAACCATTAGAGATCATAGAACAAAAACCAAAAAGAAGCAGGACTGTGAAGACAAGAGCAAGCAGGTAAGCAGCACTAGCCCCATCT
	AHCTF1_36.1_1;TTCCTACTTGACTGTATCTTTCTTTCAGAAACACAGGAAAAGGAAGTTCTTGGTCACCTCCTCCTGTAGAAATTAAGCTGGTTTCTCCCTTGGCGAGTCCAGTGGATGAAATAAAGACCG
	AHCTF1_36.1_6;TTGATTATTTTTAACATTGCTGTTTTATAATGTATGCACTTTGATGCTGTTCCTCGGATCTCTGGTCAGTACCTGTGCTCATTGGGCTTTGCCTGATACTGTGTTGAATTTGAATTGTGG
	AHCTF1_36.1_7;GCATGTGATTTTGCCTTTTTGTAGTTTTACAAATGTTGTGTAATCTACCTCAAATGAGTAATTTTCCAGTACTGCATCGGTTTCCTAGCTAGCAGTTCTCTTCAGTAACTTAAGGACTAT
	AHCTF1_36.1_8;CTTGTCTCTCAGTTCAGAGACTTAATTATCCAGGTTAGATTGACCGGTTTCACTGCTTCCTAGCAACCTCATGGAAGGATTGAAGAAAGAAATAAAACAGTGCACCGACGTGTGCCTGCT
	AHCTF1_36.1_9;GCTGAGAAGAGGAGCCGACAAGGTTTAGACTTCCCATGCTGTGAAAGAGCTTGCGACTTCGTCACAGGAGCCTCTCTAGTATTCAGGTTTAAACTGGTAACTGCCACAGAACCTCAGGC
	AHCTF1_36.1_10;ACAGGTTACAGAGAATGGAAGAGTGTGAGCAGATGTCGGAAAAGAGGAAAGGAACCCTCTTTTCTGCAGCGTGTGTACCGTTGGTAATGGCGCCTGATGGAAGCCATGATGTTCATCAAT
	AHCTF1_36.1_11;AAATAAGTGTTGTCAAATAATTTATGACAGGTAATTGATAGTGTGTAATGAATGGACGCTTAATAATTGATACCTAAAAGCAGACTTTGTCAGCTAAGCTTTGGATGTTTTCGTGAGCAG
	AHCTF1_36.1_12;TGTGCTTTGTGTGTTATTTTTCTACCGAGTAGCATCATGGATAATGAGAAAATGGGGAGAGTGCCTGTGCCGAGATCGGACAGTGTGAGAGTGGCCGTCCGGGTGCGGCCGTTCAGCCAG
	AHCTF1_36.1_13;GTCAGTCCATGCTCATTGCCTTGCTGACAATTACTGCCTGATTTTATTAATTTTTTAATAATAGTTTCTGAATTTGTATTTAAGCTGCTTGTCATTTATGAAATATTAAACTTATTTAAA
	AHCTF1_36.1_14;TGGATTTGTCAAATGATTCACAAAAGCTCTAAGCATAATTTAGTAAACATTTAATTTCATTCCTTGGTACAAGTAGCACAATAAAAAAAACTGACAAGAGAAATACTGTCAAGTTAATAA
	ENDOV_3_1;GAACAGGTGGTTGCAGCCAGCATCTGACCTGTCCCTGCTGCCTCGCTGTCAGGTGGTGTATGAGGACAGCCGCATGGTTGGCCTGAAGGCCCCCTATGTGTCAGGCTTCCTGGCCTTCCG
	ENDOV_3_2;AGAGGTCCCTTTCCTGGTGGAGTTGGTACAGCGGCTGCAAGAGAAGGAACCAGATCTCATGCCCCAGGTAGGTGCCTCCTAAGGTGAGGAGAACCCCTCTGCTTGTGCTTAGGGAGGGAA
	ENDOV_5_1;GCAGGCTTCGGGGTGGCCTGCCACCTTGGTGTCCTTACAGAGCTGCCATGCATCGGGGTGGCCAAGAAGCTCCTGCAGGTGGATGGACTGGAGAACAATGCTCTGCACAAGGAGAAGGTG
	ENDOV_6.1_1;TTTACAGTTTTTTTCTGTTTCCCAGATTGTGCTCCTGCAGGCCGGAGGAGACACATTTCCTCTGATAGGCAGCTCTGGGACTGTCCTGGGAATGGTGAGTGGCCAGAAAGCTGTGCCTTG
	ENDOV_7.1_1;ACATCCTCACAGCCTCAGTCCTGAGGCTGGTCCTGCTCCACAGCAACGTTGCCCATCCATGCTTGTCCTCCTTCCACAGGCCCTGAGGAGCCATGACCACAGCACCAAGCCCCTCTATGT
	ENDOV_7.1_2;CTCTGTGGGCCACAGAATAAGCCTGGAGGTCGCTGTGCGCCTCACCCACCACTGCTGTAGGTTCCGGATCCCAGAACCTATACGCCAGGTATGGGGCTGTGGTGGGAGTGGGAGAGTTG
	ENDOV_9.1_1;TCGGCCTTCCCTCTAGGCTGGGCCCTTCATCAGGCCTCAGAGACCCCTGGAGAACAAGGGGACTTCTGACACACCCTGGCTTTTTATGTTTCCTGTTTCTTGGTGTCATTACCAGGAGCC
	ENDOV_9.1_3;GCTTCCAGGAGCAGAAGGACCAGCAGTTGGAGGGAACCGGGCATCAGGAAGACTCGGACCTCTGGCCTCCTTCTCCAGCCTGGGTACAGTCACCACCCTGAGAAGATGATCTCTAGGAGC
	ENDOV_9.1_5;AGCCTCCTCCATCACTGGCTAGTCAGTGTGGTCACCATGCCCCAGCAGGACCATATTCCAGACCAGGAAGCACACACAAGCACCAAGAGTACATGAAGGACTTCAGGCCTCGGGTGCCCA
	ENDOV_9.1_7;AGACTGTCATCCGTGTCATAGCCAGGCTGCAGCCCCCAAGGTGCCTGTCCTCAAGCTTGTTTGCCGCTGAGCTTGAGGGACCTGCAGTTGGGAAAGCCTCAATCCTGGAGCAGGCTCAAG
	ENDOV_9.1_9;GCGTCAAAATCCCATCCCACTAAGAAGGCGGGTCTGTTCTAGCCTGGTGGCTTCTTGTCTGGGTGGCCTTGAAGGGGCTGCTGAGGCAGGCCAACAGCAGGGCTGCAGTGCCCTCCCTGT
	ENDOV_9.1_11;AGATGGTGGGGAAGCTAGCTACTAAGTCCTGTGGATACCCACAGGGGGTGGTCAGGATGGGATGACGGTCGGTGATGGGGAAGGACAGACGGGTGGTTCTCCTGTGATACCCAGGCCCAT
	ENDOV_9.1_13;TTTCAGGTTTTGGATATAAGGAAGATAGATATGTGGTTCTGTCACCCAGTAAATGAAGTGTGGGGCAGGAGGCAGCCTCCCCACCACACACCTGGTATGTATAGGCCAGCCTCCATGGGC
	ENDOV_9.1_14;AAGAACCCCACGGCCACCCAGGGTGTCCGAGCAGGACATTCCTGCAGTGTCCACATCCACCCACTGGCCCTCCGCAGTCTCTCACAAACATTCTGTGCACACACACACAACCACACATA
	ENDOV_9.2_1;CACTCTCTCTCTCTCTCTCTTCATATTCCCTGACACTCTTGTGGAAGCCAGAGCTGGAGGGCCTACAGCTGGCCCCACCATCTGCCTTCCCAACTTCCAGACCTCTCCCTTCTCAAGCCC
	ENDOV_9.2_3;CAGCCGAGCTTGAGGGCCAGCACACAGCATCCTTGTTTGAAGGGAAGCTGTTTAGAGAAGTAGGGCCCCAGAGGCCAAGGCCATCAGTCCTTAAGACCATCCTCACAGATGTGGAGGAAA
	ENDOV_9.2_5;TCAACTCTTAACAGAAGACCTTCCCCAAGACACAGAAGTCAGGCCTGATGCTGCTCAGCTTGGCACCTTCCAACACTCAGACTCTCCTCAGCCTGGGCAGAGTAGCTCTCCAGGCTGCTG
	ENDOV_9.2_6;GCTGTACAGGCAGACACTGCCAGGGGCCCACTCCCCACATACTGAGGAGCAGGGCAGAACCTGAACGTGGCCTTCTAGACAACTTTTATGTGATTTGTTCTGAAAGAAAATCCTTTCAGT
	ENDOV_9.2_9;CAAGGGCCTGTCAGCCCAGCTGCCCCGCTGGGTGTGTGTGGCAGCCCAGATTTTACCTGGCTGAGCTGCTACAGGGAGCTAAGATAGTGAGGTGCCAGGGCCAGGGGGATGCAAACTGAA
	ENDOV_9.2_10;TGAAGGAGCAGGGTGGATAGAACATAGAGTGTGGGCAAGTCTGGGGTGGGGCGTTCAGACACCCCAGACACCGGTGCTTGAGGGCAGGGGGCCCTAAGTGCAGTGGCGTCTCAGCTCCTT
	ENDOV_9.2_12;ATTTTCATATTCATCTGCAGTTTCTGTGCCCCTTCCCAGCATCCCTACCCCTGTCCGGCAGCTGGGAGAAGTTGGCTTCCAGGGGGAGGCAACCTAAACTAGCTGCTGTGTCTGTCACTC
	ENDOV_9.2_14;AAGCCATCATGGTTCCTACATACCAGACAGGCCAGGCTAGGGAACCTTGACCCTTCCTCAACACCTCAAGAGAGCCAATTGCTGTCTCCTGGCTTCTGGGGAGCCCATATGCTCCTTAGA
	ENDOV_9.2_15;TAGACCCCAATGGGAGCATACTGTCCTGCCTCCTCAGAAGAGTCCTTGAAGGGCTAGGTCTCGGGTTGAGACCTGGGGATTGCATCTGTACAGGGCTCCAGCCCCAGGTCTCTG
	ENDOV_9.3_1;GGTGTAGTGTCTCCAGAGTCAGCAGGATATCAGCCTGGACCACCTTCCCCTCCCGAGAGTGCCAGGGTGAACACCCCCCCCCAAAAACCCACTTGTCAGCCAAAGATCTGGTATCGAAG
	ENDOV_9.3_3;ATCCCCACTGCACTGCCCTAACCAATTGATTAATTAAAAGCAGAGGCACAGAACAGCGGCACGGGACCACTGACTTGCAGATGAATGGCCCAAGAAGTAGTCAGGTGGGTGTCGCCGTGG
	ENDOV_9.3_5;AATGGGAAGATAAAGTGCAGGAGCCGGGACTCCAGGCAGAGGCACCTGGATCAGGTCTTGATGGAGAGGGTGTAGTTTGGGCTTGGGCTGGGATTTTGTCTTGTTTTGTTTTAACAGGGT
