# Supplementary material for: CRISPR-Mediated Knockout of Long 3′ UTR mRNA Isoforms in mESC-Derived Neurons
Source: Front Genet. 2021 Dec 17;12:789434. doi: 10.3389/fgene.2021.789434 (PMC8718760; doi:10.3389/fgene.2021.789434)
Supplement: Supplementary file 3 [file DataSheet1.PDF]

Supplementary Material

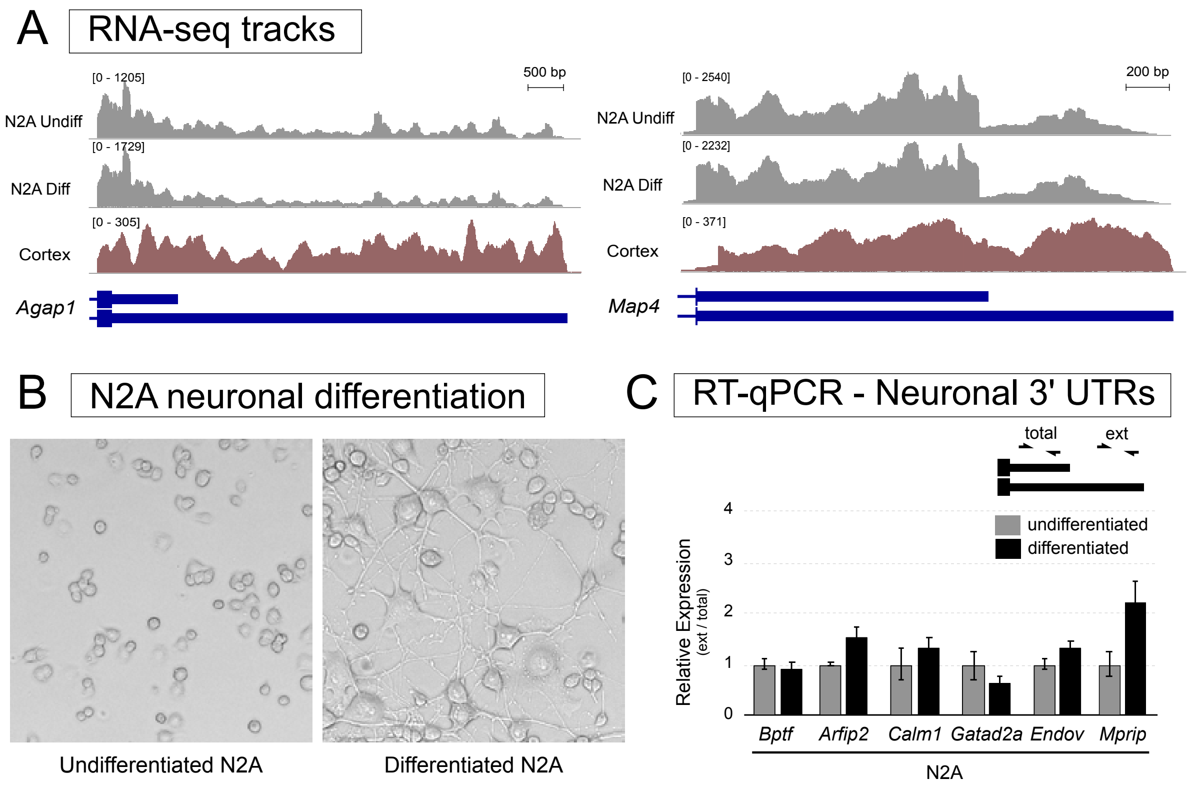

Supplementary Figure 1. Differentiation of Neuro2a cells. (A) RNA-seq tracks for two APA genes show long 3' UTR isoforms are not upregulated by N2a differentiation (grey tracks). Note the robust expression of long 3' UTRs in the cortex tracks. **(B)** Images of undifferentiated and 7 day differentiated N2a cells. Morphological changes suggest successful differentiation. **(C)** RT-qPCR shows lack of long 3' UTR isoform upregulation in differentiated N2a cells. These same 6 genes displayed increased long 3' UTR expression in mESC-derived neurons (see **Figure 1E**). Two-tail t-test were performed between the undifferentiated and differentiated cells, and no significant changes were observed.

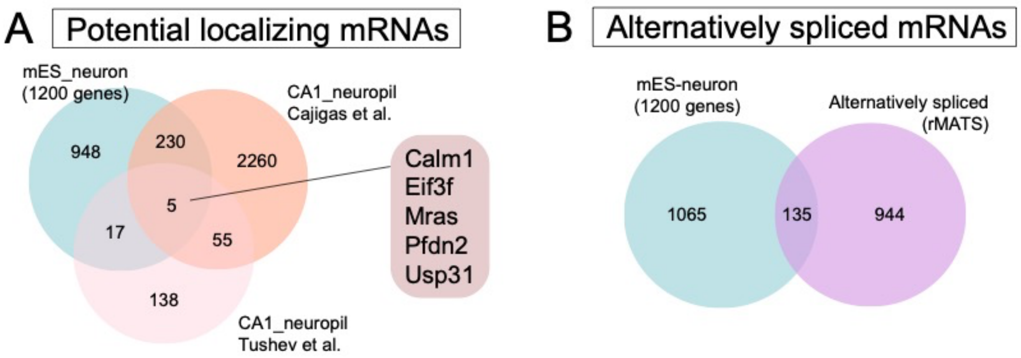

Supplementary Figure 2. Overlap of 1200 3' UTR lengthening genes with lists of neuropil localized and alternatively spliced genes. (A) The list of 1200 genes that undergo lengthening of 3' UTR during the mESC neuronal differentiation (**Figure 1B**) was compared with previously reported lists of genes expressing mRNAs localized to hippocampal neuropil. Overlap of 252 3' UTR lengthening genes with at least one of the subcellular localization lists was found, and 5 genes were found to overlap with both subcellular localization lists. **(B)** Alterative splicing analysis was performed using rMATS using the RNA-seq dataset (splicing change $\Delta PSI > 0.2$; $FDR < 0.05$). A list of genes that presented both alternative polyadenylation and cassette exon alternative splicing was obtained by overlapping the rMATS list and the 1200 lengthening genes (**Figure 1B**). This analysis revealed 135 genes subject to both alternative splicing and alternative polyadenylation during mESC neuronal differentiation.
